# The dose-response effect of aerobic exercise on inflammation in colon cancer survivors

**DOI:** 10.3389/fonc.2023.1257767

**Published:** 2023-12-12

**Authors:** Justin C. Brown, Stephanie L.E. Compton, Jeffrey A. Meyerhardt, Guillaume Spielmann, Shengping Yang

**Affiliations:** ^1^ Pennington Biomedical Research Center, Baton Rouge, LA, United States; ^2^ Louisiana State University (LSU) Health Sciences Center New Orleans School of Medicine, New Orleans, LA, United States; ^3^ Stanley S. Scott Cancer Center, Louisiana State University Health Sciences Center, New Orleans, LA, United States; ^4^ Louisiana Cancer Research Center, New Orleans, LA, United States; ^5^ Dana-Farber Cancer Institute, Boston, MA, United States; ^6^ School of Kinesiology, College of Human Sciences & Education, Louisiana State University, Baton Rouge, LA, United States

**Keywords:** biomarkers, C-reactive protein, dose-response, interleukins, tumor necrosis factors

## Abstract

**Background:**

Physical activity after surgical resection for colon cancer is associated with significantly longer disease-free survival. Inflammation is hypothesized to mediate the association between physical activity and disease-free survival in colon cancer.

**Methods:**

In this exploratory analysis of a randomized dose-response trial, 39 colon cancer survivors who completed standard therapy were stratified by cancer stage and randomized in a 1:1:1 ratio to one of three treatment groups for 24 weeks of usual-care control, 150 min/wk of moderate-intensity aerobic exercise (low-dose), or 300 min/wk of moderate-intensity aerobic exercise (high-dose). Inflammation outcomes included high-sensitivity C-reactive protein (hs-CRP), interleukin-6 (IL6), and soluble tumor necrosis factor-alpha receptor 2 (sTNFαR2). Mixed models for repeated measures were used to test the hypothesis that exercise was associated with dose-response reductions in inflammation; exploratory analyses examined treatment effects by cancer stage.

**Results:**

In the overall population, aerobic exercise was not associated with dose-response reductions in hs-CRP, IL6, or sTNFαR2. Cancer stage modified the association between randomized group and hs-CRP (P=0.022) and IL6 (P<0.001) but not sTNFαR2 (P=0.39). In stage I-II disease, compared to control, exercise was not associated with inflammation outcomes. In stage III disease, compared to control, low-dose exercise reduced hs-CRP: −35.4% (95% CI: −70.1, −0.7) and IL6: −29.6% (95% CI: −58.4, −0.8) but not sTNFαR2: 2.7% (95% CI: sTNFαR2: −15.7, 21.1); high-dose exercise was not associated with inflammation outcomes in stage III disease.

**Conclusion:**

This exploratory analysis offers preliminary data to support the hypothesis that inflammation may mediate the association between physical activity and disease-free survival in colon cancer.

**Clinical trial registration:**

clinicaltrials.gov, identifier NCT02250053.

## 1 Introduction

Physical activity after surgical resection for colon cancer is associated with a significantly longer disease-free survival (1, 2), by reducing the risk of disease recurrence in a subset of patients (3). The association between physical activity and disease-free survival is independent of known prognostic factors and occurs in a dose-response fashion, such that larger volumes of physical activity are associated with a higher probability of remaining alive and cancer-free (4). The biological mechanisms by which physical activity is associated with improved disease-free survival remain incompletely understood, but inflammation is postulated as a key mediator (5).

Inflammation activates the JAK-STAT and NF-κB signaling pathways to promote cancer cell proliferation, migration, and invasion (6). Inflammation that persists after recovery from colonic tumor resection is independently associated with shorter disease-free survival (7–9). In animal models, experimental manipulation of inflammatory pathways regulates the growth and progression of colonic tumors (10). However, data from clinical trials in colon cancer survivors are limited (11), and the effects of different exercise doses on inflammation outcomes are unknown.

We conducted an exploratory analysis to examine the effects of distinct doses of aerobic exercise using data from a trial that randomized colon cancer survivors to one of three groups for 24 weeks: usual-care control, 150 min/wk of moderate-intensity aerobic exercise (low-dose), or 300 min/wk of moderate-intensity aerobic exercise (high-dose) (12). We hypothesized that exercise would reduce inflammation in a dose-response fashion. Inflammation may correlate with colon cancer disease stage (13). Therefore, we examined if subjects with higher-stage colon cancer derive a larger anti-inflammatory benefit from exercise.

## 2 Methods

### 2.1 Study design

This study was a 24-week, phase II, single-center, randomized, dose-response trial. The study followed Good Clinical Practice and the ethical principles in the Declaration of Helsinki. The Institutional Review Board approved the trial protocol and informed consent document. All subjects provided informed consent and approval from their physician before completing any study activities. The study was registered on clinicaltrials.gov as NCT02250053, and detailed study methods are published (12). The prespecified primary and secondary outcomes are published (14–18). The inflammation outcomes reported here were not prespecified and were conducted for exploratory purposes to inform the design of future studies.

### 2.2 Subjects

Subjects were eligible if they were diagnosed with histologically-proven stage I-III colon cancer, underwent surgical tumor resection, completed postoperative chemotherapy within 36 months of entering the study (if applicable), self-reported <150 min/wk of moderate- to vigorous-intensity physical activity (19), were age ≥18 years, provided written physician approval, had no additional surgery planned within the 24 week intervention period, and could walk unassisted for six minutes.

### 2.3 Randomization and blinding

Subjects were stratified by cancer stage (I vs. II vs. III) and randomized to one of three groups: usual-care control, 150 min/wk of moderate-intensity aerobic exercise (low-dose), or 300 min/wk of moderate-intensity aerobic exercise (high-dose). Subjects were not blinded to treatment assignment. Outcome measures were obtained by assessors blinded to treatment assignment.

### 2.4 Intervention

Subjects randomized to the low-dose or high-dose exercise groups utilized a study-provided in-home treadmill and heart rate monitor. The exercise intensity was prescribed at 50−70% of the age-predicted maximum heart rate. The low-dose and high-dose target exercise volume was 150 and 300 min/wk, respectively. Subjects were encouraged to individualize their frequency (days per week), fractionation (sessions per day), and duration (minutes per session) of exercise according to a schedule that promoted a high level of adherence to the prescribed exercise volume (17). Subjects randomized to the usual-care control group maintained their pre-study physical activity levels.

### 2.5 Measurements

Demographic characteristics, including age, sex, and race, were self-reported. Cancer stage was obtained from the cancer registry, pathology reports, and physician records. At baseline and week 24, subjects underwent a fasting blood draw. Blood draws were performed after a minimum eight-hour fast and abstinence from alcohol consumption for 24 hours. A total of 30 mL of plasma was centrifuged, aliquoted, and stored at −80°C following standardized procedures. Circulating tumor cells were measured as previously described (15).

### 2.6 Inflammatory outcomes

Inflammation measures included high-sensitivity C-reactive protein (hs-CRP), interleukin 6 (IL6), and soluble tumor necrosis factor-alpha receptor 2 (sTNFαR2). These inflammatory measures are associated with disease-free survival in colon cancer survivors (20–22). hs-CRP was measured as a marker of overall systemic inflammation (23). IL6 was measured as an activator of the JAK-STAT pathway (24). sTNFαR2 was measured as an activator of the NF-kB pathway (25). sTNFαR2 is a surrogate marker for TNFα that is more stable in plasma and less sensitive to diurnal variation (26). hs-CRP was quantified using an immunoturbidimetric assay (Roche Diagnostics). IL6 and sTNFαR2 were quantified using ultrasensitive sandwich enzyme immunoassays (R&D Systems). Baseline and week 24 samples were assayed simultaneously and in duplicate at the end of the study. Blinded quality-control samples were interspersed among cases. The median [interquartile range] time from biospecimen collection to laboratory analysis was 3.7 years [3.4, 3.8], and all samples were never previously thawed (27). The coefficients of variation for all samples were ≤8% (11).

### 2.7 Statistical analyses

Descriptive statistics for baseline variables include counts and proportions for categorical variables and means and standard deviations for continuous variables. Dependent variables were log-transformed in the inferential analysis to improve the distributional normality (28). The change was evaluated from baseline to follow-up in the three groups using mixed models for repeated measures. This modeling technique includes all data and accounts for the correlation between measures. Treatment effects were calculated as the treatment effect ratio, which quantifies the percent change in geometric means from baseline to week 24 (e.g., a treatment effect ratio of 0.75 indicates a 25% reduction), with 95% confidence intervals (CI). The regression models included the baseline value of the dependent variable and cancer stage (randomization stratification factor) as covariates to improve the precision of effect size estimation (29). Group-by-time interaction terms were included as fixed effects in the regression model. A test of trends with linear and nonlinear (quadratic) contrasts evaluated the presence of a dose-response relationship across randomized groups. Effect modification by cancer stage was evaluated by including a three-way interaction among cancer stage, randomized group, and time in the mixed models for repeated measures.

At randomization, cancer stage was a three-level variable (I vs. II vs.. III). However, for this analysis, subjects with stage I and II disease were combined (n=19) to provide a numeric balance to the number of subjects with stage III disease (n=20). The threshold for statistical significance for interactions was prespecified at P<0.10, because of known limitations in statistical power (30). Model fit was assessed using graphical and numeric techniques. Stata 17.0 (College Station, TX, USA) was used for all statistical analyses, and GraphPad Prism 9.4 (Boston, MA, USA) for data visualization.

## 3 Results

Subjects were recruited and randomized between January 2015 and August 2015, with data collection ending in February 2016. The study completion rate was 97% (one subject was lost to follow-up; **Supplementary Figure 1**). At baseline, the age ranged from 35 to 81 years, and subjects were most often female (62%), of white race (79%), with stage III disease (51%), and treated with chemotherapy (72%; **Table 1**).

**Table 1:** Baseline subject characteristics overall and by randomized group.

Characteristic	Overall (n=39)	Control (n=13)	Low-Dose (n=14)	High-Dose (n=12)
Age, years	56.5 ± 10.0	57.9 ± 9.7	58.2 ± 9.8	53.1 ± 10.5
Sex, %				
Male	15 (38%)	4 (31%)	7 (50%)	4 (33%)
Female	24 (62%)	9 (69%)	7 (50%)	8 (67%)
Race, %				
White	31 (79%)	8 (62%)	12 (86%)	11 (92%)
Nonwhite	8 (21%)	5 (38%)	2 (14%)	1 (8%)
Cancer Stage, %				
I/II	19 (49%)	6 (46%)	7 (50%)	6 (50%)
III	20 (51%)	7 (54%)	7 (50%)	6 (50%)
Chemotherapy, %				
Yes	28 (72%)	10 (77%)	10 (71%)	8 (67%)
No	11 (28%)	3 (23%)	4 (29%)	4 (33%)

Data are mean ± standard deviation or n (%).

At baseline, the mean (SD) hs-CRP was 2.53 (2.11) mg/L, IL6 was 2.07 (1.63) pg/mL, and sTNFαR2 was 2524 (840) pg/mL, indicating low to moderate inflammation. From baseline to week 24, the low-dose and high-dose groups completed an average of 141 min/wk (93% adherence) and 247 min/wk (89% adherence) of exercise, respectively. Exercise adherence ranged from 17−100% and 21−100% in the low-dose and high-dose groups, respectively. The low-dose and high-dose exercise groups averaged 3.5 and 4.3 days of exercise each week and 41.6 and 59.1 minutes per session, respectively. Detailed adherence trajectories have been reported (17).

In the overall intention-to-treat population, randomization to higher doses of aerobic exercise was not associated with dose-response reductions in hs-CRP (linear P=0.74; nonlinear P=0.41), IL6 (linear P=0.11; nonlinear P=0.77), or sTNFαR2 [(linear P=0.66; nonlinear P=0.75); **Table 2**].

**Table 2:** Change in inflammation outcomes by randomized group.

Inflammation outcome	Randomized group	Baseline geometric mean (SD)	Geometric mean change (SE)	Intervention main effect, treatment ratio (95% CI)	Percent difference between groups (95% CI)
hs-CRP, mg/L	Control	0.07 (1.26)	0.10 (0.14)	1.00 (Reference)	0.00 (Reference)
	Low-Dose	0.60 (1.23)	−0.01 (0.13)	0.90 (0.56, 1.25)	−9.8 (−44.0, 24.5)
	High-Dose	0.58 (1.10)	0.16 (0.14)	1.07 (0.65, 1.49)	6.8 (−35.2, 48.9)
IL6, pg/mL	Control	0.60 (0.60)	−0.23 (0.13)	1.00 (Reference)	0.00 (Reference)
	Low-Dose	0.41 (0.65)	−0.04 (0.11)	1.21 (0.80, 1.62)	20.7 (−20.3, 61.7)
	High-Dose	0.52 (0.75)	0.06 (0.12)	1.34 (0.87, 1.81)	33.6 (−13.4, 80.7)
sTNFαR2, pg/mL	Control	7.65 (0.29)	−0.06 (0.04)	1.00 (Reference)	0.00 (Reference)
	Low-Dose	7.89 (0.27)	−0.03 (0.04)	1.03 (0.91, 1.15)	3.1 (−9.2, 15.4)
	High-Dose	7.79 (0.38)	−0.03 (0.04)	1.03 (0.90, 1.16)	2.8 (−9.9, 15.5)

SD, standard deviation; LS Mean, least squares mean; SE, standard error; CI, confidence interval. Models are adjusted for cancer stage (randomization stratification factor) and baseline value of the dependent variable.

At study enrollment, subjects with stage I or II colon cancer had completed cancer-directed treatments a mean of 12.0 (5.6) months previously, and subjects with stage III colon cancer completed cancer-directed treatments a mean of 9.0 (6.1) months previously [Δ: −2.9 months (95% CI: −5.6, −0.3)]. Subjects with stage I and II colon cancer did not have different concentrations of hs-CRP (P=0.26), IL6 (P=0.74), or sTNFαR2 (P=0.44). Cancer stage modified the association between randomized group and hs-CRP (P_interaction_=0.022) and IL6 (P_interaction_<0.001) but not sTNFαR2 (P_interaction_=0.39). Exercise adherence did not differ between subjects with stage I or II versus stage III colon cancer (P=0.17).

Compared to control, randomization to low-dose or high-dose aerobic exercise was not associated with inflammation outcomes in subjects with stage I or II colon cancer (**Table 3**). Conversely, compared to control, randomization to low-dose aerobic exercise statistically significantly reduced hs-CRP: −35.4% (95% CI: −70.1, −0.7) and IL6: −29.6% (95% CI: −58.4, −0.8), but not sTNFαR2: 2.7% (95% CI: −15.7, 21.1) in subjects with stage III cancer, whereas randomization to high-dose aerobic exercise was not associated with a reduction in any inflammation outcome in subjects with stage III colon cancer.

**Table 3:** Change in inflammation outcomes by cancer stage subgroup and randomized group.

Inflammation outcome	Cancer stage subgroup	Randomized group	Baseline geometric mean (SD)	Geometric mean change (SE)	Intervention main effect, treatment ratio (95% CI)	Percent difference between groups (95% CI)	P cancer stage-by-group-by-time interaction
hs-CRP, mg/L	Stage I-II	Control	1.00 (0.29)	0.11 (0.15)	1.00 (Reference)	0.00 (Reference)	0.022
		Low-Dose	0.35 (1.49)	0.37 (0.11)*	1.30 (0.82, 1.78)	29.6 (−18.4, 77.06)	
		High-Dose	1.14 (0.85)	0.33 (0.12)*	1.24 (0.77, 1.71)	24.0 (−23.3, 71.3)	
	Stage III	Control	−0.60 (1.27)	0.05 (0.19)	1.00 (Reference)	0.00 (Reference)	
		Low-Dose	0.84 (0.96)	−0.38 (0.19)*	0.65 (0.30, 0.99)	−35.4 (−70.1, −0.7)	
		High-Dose	0.01 (1.08)	−0.01 (0.21)	0.95 (0.42, 1.48)	−5.3 (−58.2, 47.6)	
IL6, pg/mL	Stage I-II	Control	0.97 (0.48)	−0.49 (0.15)*	1.00 (Reference)	0.00 (Reference)	<0.001
		Low-Dose	0.29 (0.52)	0.35 (0.11)*	2.33 (1.47, 3.18)	132.6 (47.2, 218.1)	
		High-Dose	0.74 (0.84)	0.14 (0.12)	1.89 (1.17, 2.60)	88.9 (17.3, 160.4)	
	Stage III	Control	0.33 (0.56)	−0.08 (0.15)	1.00 (Reference)	0.00 (Reference)	
		Low-Dose	0.53 (0.78)	−0.43 (0.15)*	0.70 (0.42, 0.99)	−29.6 (−58.4, −0.8)	
		High-Dose	0.30 (0.66)	−0.02 (0.16)	1.06 (0.61, 1.52)	6.4 (−38.9, 51.7)	
sTNFαR2, pg/mL	Stage I-II	Control	7.64 (0.22)	0.02 (0.05)	1.00 (Reference)	0.00 (Reference)	0.39
		Low-Dose	7.85 (0.29)	0.02 (0.04)	1.00 (0.86, 1.14)	0.0 (−13.6, 13.7)	
		High-Dose	7.65 (0.27)	−0.01 (0.05)	0.97 (0.83, 1.10)	−3.3 (−16.9, 10.4)	
		Control	7.65 0.35)	−0.11 (0.06)	1.00 (Reference)	0.00 (Reference)	
	Stage III	Low-Dose	7.96 (0.27)	−0.08 (0.06)	1.03 (0.84, 1.21)	2.7 (−15.7, 21.1)	
		High-Dose	7.93 (0.43)	−0.05 (0.07)	1.06 (0.86, 1.25)	5.6 (−14.1, 25.3)	

SD, standard deviation; LS Mean, least squares mean; SE, standard error; CI, confidence interval. Models are adjusted for the baseline value of the dependent variable.

"*" P<0.05 (within group).

**Figure 1:**
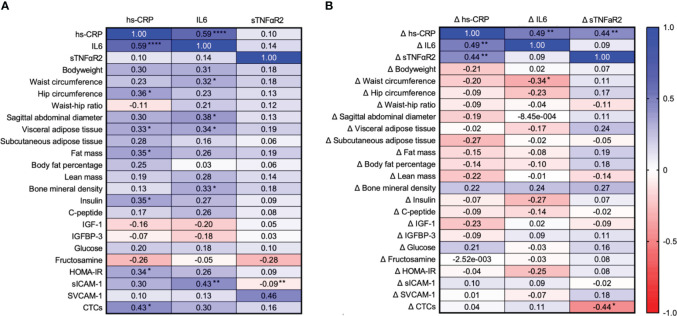
Correlational analyses of inflammation outcomes at baseline **(A)** and change from baseline to week 24 **(B)** with previously reported variables are presented for hypothesis generation hs-CRP, high sensitivity C-reactive protein; IL6, interleukin 6; sTNFαR2, soluble tumor necrosis factor alpha receptor 2; IGF-1, insulin-like growth factor 1; IGFBP-3, insulin-like growth factor binding protein 3; HOMA-IR, homeostatic model of insulin resistance; sICAM-1, soluble intercellular adhesion molecule 1; sVCAM-1, soluble vascular adhesion molecule 1; CTCs, circulating tumor cells. *P<0.05; **P<0.01; ***P<0.001.

Correlational analyses of inflammation outcomes at baseline and change from baseline to week 24 with previously reported variables are presented for hypothesis generation (**Figure 1**). Notably, baseline hs-CRP correlated with circulating tumor cells (r=0.43; 95% CI: 0.07, 0.68), and the change from baseline to week 24 in sTNFαR2 correlated with the change in circulating tumor cells (r=−0.44; 95% CI: −0.72, −0.04).

## 4 Discussion

In this exploratory analysis of insufficiently physically active colon cancer survivors with low to moderate inflammation at baseline, randomization to 150 min/wk of moderate-intensity aerobic exercise for 24 weeks reduced concentrations of hs-CRP and IL6 in those with stage III disease. Randomization to 300 min/wk of moderate-intensity aerobic exercise was not associated with any inflammation-lowering effect, nor was either dose of aerobic exercise assigned to those with stage I or II colon cancer. In correlational analyses, inflammation was associated with circulating tumor cell burden at baseline and during follow-up.

One mechanism by which physical activity is hypothesized to exert anticancer effects is by reducing inflammation within the host microenvironment (5). Our results demonstrate that 24 weeks of 150 min/wk of moderate-intensity aerobic exercise reduce hs-CRP and IL6 by 35.4% and 29.6% among stage III colon cancer survivors. In a prospective cohort study of 1,494 stage III colon cancer survivors, elevated hs-CRP and IL6 were associated with a 65% and 52% higher relative risk of disease recurrence or death, respectively (9). Our findings are consistent with the hypothesis that inflammation is a key mediator of the association between physical activity and disease-free survival in colon cancer survivors. Moreover, our results enhance the clinical relevance of experiments in tumor-bearing preclinical models that conclude inflammatory pathway blockade slows cancer cell growth and delays tumor progression (31, 32).

The results of the current analysis complement a prior trial that was conducted as part of the National Cancer Institutes (NCI) Transdisciplinary Research on Energetics and Cancer (TREC) Consortium (33). This prior trial used a 2×2 factorial design to evaluate the effect of 12 weeks of exercise or metformin on inflammation in 139 breast and colorectal cancer patients (11). Compared with control, randomization to 220 min/wk of moderate-intensity aerobic exercise statistically significantly reduced hs-CRP: −30.2% (95% CI, −50.3, −1.0) and IL6: −30.9% (95% CI, −47.3, −9.5); but did not significantly change sTNFαR2: 1.0% (95% CI, −10.4, 13.9) (11). Our results are compatible regarding the specificity of inflammatory biomarker response (e.g., hs-CRP and IL6 were lowered with exercise but not sTNFαR2) and the magnitude of biomarker response (e.g., −35.4% vs.. 30.2% for hs-CRP and −29.6% vs.. −30.9 for IL6). The absence of a statistically significant dose-response effect in the current analysis may indicate that the optimal dose of moderate-intensity aerobic exercise to reduce the studied inflammatory biomarkers in colon cancer survivors is between 150 and 220 min/wk.

The current analysis results complement what is known in healthy subjects without a history of cancer. In the Alberta Physical Activity and Breast Cancer Prevention (ALPHA) Trial, 320 postmenopausal women were randomized to 52 weeks of aerobic exercise or a usual care control group. Compared to control, randomization to 225 min/wk of moderate- to vigorous-intensity aerobic exercise statistically significantly reduced hs-CRP: −13% (95% CI: −21, −4), but did not significantly change IL6: −1% (95% CI: −8, 7) or TNFα: 0% (95% CI: −3, 4) (34). The Breast Cancer and Exercise Trial in Alberta (BETA) randomized 400 postmenopausal women to 52 weeks of 150 min/wk or 300 min/wk of aerobic exercise. Compared to 150 min/wk, randomization to 300 min/wk of moderate- to vigorous-intensity aerobic exercise, did not significantly change hs-CRP, IL6, or TNFα (35). The effects of exercise on inflammation in subjects without cancer has been summarized in a meta-analysis (36). These data in subjects without cancer are comparable to those in cancer survivors, such that the dose-response curve between exercise volume and change in inflammatory outcomes is not linear.

Our hypothesis that patients with higher-stage colon cancer derive a larger anti-inflammatory benefit from exercise was supported. Although our hypothesis was supported, subjects with stage III disease did not have more inflammation than subjects with stage I-II disease. This contrasts with prior reports that inflammation correlates with colon cancer disease stage (13). Aside from the extent of invasion through the bowel wall (T-stage) and lymph node metastases (N-stage), which are used to determine the American Joint Committee on Cancer (AJCC) overall cancer stage (37), the only baseline factor that differed between stage I-II versus stage III colon cancer survivors was the receipt of chemotherapy. However, chemotherapy per se did not modify the association between randomized groups and inflammatory outcomes. Other factors measured after randomization, such as exercise adherence, did not differ between subjects with stage I-II versus stage III disease. The biological explanation of why cancer stage modifies the association between randomized groups and inflammatory outcomes, therefore, remains uncertain. This observation will be prospectively interrogated in an ongoing randomized trial of exercise in colorectal cancer survivors (e.g., NCT03975491).

We previously reported that exercise lowers circulating tumor cells using this dataset. Over 24 weeks, statistically significant decreases in circulating tumor cells were observed in the low- and high-dose exercise groups, whereas no significant change was observed in the control group (15). Anthropometric measures, such as visceral fat, and metabolic measures, such as fasting insulin, were biological mediators of the association between exercise and reductions in circulating tumor cells (15). The current analysis suggests inflammation is a potential biological mediator of the association between exercise and reductions in circulating tumor cells. In a cross-sectional study of women with metastatic breast cancer, circulating tumor cells positively correlated with CRP (r=0.22; P=0.02) and IL6 (r=0.25; P=0.01) (38). Changes in circulating tumor cells after surgery and chemotherapy are prognostic of disease-free survival in colorectal cancer survivors (39, 40). Results from an ongoing randomized clinical trial (e.g., NCT03975491) will clarify the interplay of inflammation with circulating tumor cells and circulating tumor DNA to offer unique insight into mechanisms of treatment benefit in colorectal cancer survivors.

There are several limitations to this analysis. The primary limitation is that inflammatory outcomes were not prespecified in the study protocol; consequently, the results of this analysis are hypothesis-generating. The small sample size may have limited our ability to detect small but potentially clinically meaningful effects of exercise on inflammatory outcomes. The sample sizes were very small in the analysis stratified by cancer stage, resulting in uncertainty in the point estimates. The study duration was 24 weeks, which limits our ability to understand the benefits of exercise on inflammatory outcomes acutely and over longer time horizons. Study subjects were not enrolled based on having high inflammation at baseline, which limits our understanding of the effects of exercise in those with acute or chronic inflammation. We examined two distinct volumes of moderate-intensity aerobic exercise but not the effects of exercise intensity (e.g., light vs. moderate vs. vigorous) or exercise modality (e.g., weightlifting vs. high-intensity interval training) on inflammation outcomes. We examined three inflammation biomarkers associated with disease-free survival in colon cancer survivors (20–22). However, we acknowledge that inflammation can be quantified using many other biomarkers.

There are several strengths to this analysis. The two intervention groups, each prescribed a distinct dose of moderate-intensity aerobic exercise, allowed us to examine how inflammation outcomes change along the exercise dose curve. The aerobic exercise program was flexible, emphasizing home-based exercise, complemented with behavioral coaching from an exercise physiologist. Providing home-based treadmills incentivized study enrollment, as recruitment was completed ahead of schedule, and promoted excellent adherence to the exercise prescription over 24 weeks. Exercise adherence was objectively quantified using heart-rate monitors eliminating bias from self-report. Endpoint data collection, including inflammation assays, was conducted by staff blinded to the study group who adhered to standardized protocols to enhance rigor and reproducibility. Endpoint data collection was excellent (97% follow-up).

In one of the first randomized clinical trials evaluating two doses of moderate-intensity aerobic exercise in colon cancer survivors, this study suggests that 150 min/wk of moderate-intensity aerobic exercise may lower inflammation in select colon cancer survivors. The findings from this exploratory analysis are useful to inform the design of future studies that aim to identify the biological mediators of the relationship between physical activity and disease-free survival in colon cancer survivors.

## Data availability statement

The raw data supporting the conclusions of this article will be made available by the authors, without undue reservation.

## Ethics statement

The studies involving humans were approved by University of Pennsylvania School of Medicine. The studies were conducted in accordance with the local legislation and institutional requirements. The participants provided their written informed consent to participate in this study.

## Author contributions

JB: Writing – original draft, Writing – review & editing. SC: Writing – review & editing. JM: Writing – review & editing. GS: Writing – review & editing. SY: Writing – review & editing.

## References

[1] Meyerhardt JA Heseltine D Niedzwiecki D Hollis D Saltz LB Mayer RJ. Impact of physical activity on cancer recurrence and survival in patients with stage III colon cancer: findings from CALGB 89803. J Clin Oncol (2006) 24(22):3535–41. doi: 10.1200/JCO.2006.06.0863 16822843

[2] Brown JC Ma C Shi Q Fuchs CS Meyer J Niedzwiecki D. Physical activity in stage III colon cancer: CALGB/SWOG 80702 (Alliance). J Clin Oncol (2023) 41(2):243–54. doi: 10.1200/JCO.22.00171 PMC9839249 35944235

[3] Brown JC Ma C Shi Q Niedzwiecki D Zemla T Couture F. Association between physical activity and the time course of cancer recurrence in stage III colon cancer. Br J Sports Med (2023) 57(15):965–971. doi: 10.1136/bjsports-2022-106445 PMC10423490 36878665

[4] McTiernan A Friedenreich CM Katzmarzyk PT Powell KE Macko R Buchner D. Physical activity in cancer prevention and survival: A systematic review. Med Sci Sports Exerc. (2019) 51(6):1252–61. doi: 10.1249/MSS.0000000000001937 PMC6527123 31095082

[5] Brown JC Gilmore LA. Physical activity reduces the risk of recurrence and mortality in cancer patients. Exerc Sport Sci Rev (2020) 48(2):67–73. doi: 10.1249/JES.0000000000000214 31913187 PMC7071977

[6] Mantovani A Allavena P Sica A Balkwill F. Cancer-related inflammation. Nature (2008) 454(7203):436–44. doi: 10.1038/nature07205 18650914

[7] Yasui K Shida D Nakamura Y Ahiko Y Tsukamoto S Kanemitsu Y. Postoperative, but not preoperative, inflammation-based prognostic markers are prognostic factors in stage III colorectal cancer patients. Brit J Cancer. (2021) 124(5):933–41. doi: 10.1038/s41416-020-01189-6 PMC7921100 33257844

[8] Chan JCY Diakos CI Chan DLH Engel A Pavlakis N Gill A. A longitudinal investigation of inflammatory markers in colorectal cancer patients perioperatively demonstrates benefit in serial remeasurement. Ann Surg (2018) 267(6):1119–25. doi: 10.1097/SLA.0000000000002251 28394869

[9] Cheng E Shi Q Shields AF Nixon AB Shergill AP Ma C. Association of inflammatory biomarkers with survival among patients with stage III colon cancer. JAMA Oncol (2023) 9(3):404–13. doi: 10.1001/jamaoncol.2022.6911 PMC9880869 36701146

[10] Chung YC Ku YL Chiang HC Liu WC Kao TY Yang CH. Antibody to interleukin-6 receptor inhibits *in vivo* growth of human colorectal carcinoma cell xenografts. Anticancer Res (2021) 41(10):4907–16. doi: 10.21873/anticanres.15304 34593438

[11] Brown JC Zhang S Ligibel JA Irwin ML Jones LW Campbell N. Effect of exercise or metformin on biomarkers of inflammation in breast and colorectal cancer: A randomized trial. Cancer Prev Res (Phila). (2020) 13(12):1055–62. doi: 10.1158/1940-6207.CAPR-20-0188 PMC7718298 32859615

[12] Brown JC Troxel AB Ky B Damjanov N Zemel BS Rickels MR. A randomized phase II dose-response exercise trial among colon cancer survivors: Purpose, study design, methods, and recruitment results. Contemp Clin Trials. (2016) 47:366–75. doi: 10.1016/j.cct.2016.03.001 PMC4818956 26970181

[13] Kantola T Klintrup K Vayrynen JP Vornanen J Bloigu R Karhu T. Stage-dependent alterations of the serum cytokine pattern in colorectal carcinoma. Brit J Cancer. (2012) 107(10):1729–36. doi: 10.1038/bjc.2012.456 PMC3493870 23059742

[14] Brown JC Damjanov N Courneya KS Troxel AB Zemel BS Rickels MR. A randomized dose-response trial of aerobic exercise and health-related quality of life in colon cancer survivors. Psychooncology (2018) 27(4):1221–8. doi: 10.1002/pon.4655 PMC5895514 29388275

[15] Brown JC Rhim AD Manning SL Brennan L Mansour AI Rustgi AK. Effects of exercise on circulating tumor cells among patients with resected stage I-III colon cancer. PloS One (2018) 13(10):e0204875. doi: 10.1371/journal.pone.0204875 30332430 PMC6192582

[16] Brown JC Rickels MR Troxel AB Zemel BS Damjanov N Ky B. Dose-response effects of exercise on insulin among colon cancer survivors. Endocr Relat Cancer. (2018) 25(1):11–9. doi: 10.1530/ERC-17-0377 PMC5736434 29018055

[17] Brown JC Troxel AB Ky B Damjanov N Zemel BS Rickels MR. Dose-response effects of aerobic exercise among colon cancer survivors: A randomized phase II trial. Clin Colorectal Cancer. (2018) 17(1):32–40. doi: 10.1016/j.clcc.2017.06.001 28669606 PMC5733696

[18] Brown JC Zemel BS Troxel AB Rickels MR Damjanov N Ky B. Dose-response effects of aerobic exercise on body composition among colon cancer survivors: a randomised controlled trial. Brit J Cancer. (2017) 117(11):1614–20. doi: 10.1038/bjc.2017.339 PMC5729439 28934762

[19] Paffenbarger R Wing A Hyde R. Paffenbarger physical activity questionnaire. Am J Epidemiol. (1978) 108:161–75. doi: 10.1093/oxfordjournals.aje.a112608 707484

[20] Shrotriya S Walsh D Bennani-Baiti N Thomas S Lorton C. C-reactive protein is an important biomarker for prognosis tumor recurrence and treatment response in adult solid tumors: A systematic review. PloS One (2015) 10(12):e0143080. doi: 10.1371/journal.pone.0143080 26717416 PMC4705106

[21] Knupfer H Preiss R. Serum interleukin-6 levels in colorectal cancer patients–a summary of published results. Int J Colorectal Dis (2010) 25(2):135–40. doi: 10.1007/s00384-009-0818-8 19898853

[22] Babic A Shah SM Song M Wu K Meyerhardt JA Ogino S. Soluble tumour necrosis factor receptor type II and survival in colorectal cancer. Brit J Cancer. (2016) 114(9):995–1002. doi: 10.1038/bjc.2016.85 27031855 PMC4984918

[23] Black S Kushner I Samols D. C-reactive protein. J Biol Chem (2004) 279(47):48487–90. doi: 10.1074/jbc.R400025200 15337754

[24] Sansone P Bromberg J. Targeting the interleukin-6/Jak/stat pathway in human Malignancies. J Clin Oncol (2012) 30(9):1005–14. doi: 10.1200/JCO.2010.31.8907 PMC3341105 22355058

[25] Rodriguez M Cabal-Hierro L Carcedo MT Iglesias JM Artime N Darnay BG. NF-kappaB signal triggering and termination by tumor necrosis factor receptor 2. J Biol Chem (2011) 286(26):22814–24. doi: 10.1074/jbc.M111.225631 PMC3123049 21558270

[26] Chan AT Ogino S Giovannucci EL Fuchs CS. Inflammatory markers are associated with risk of colorectal cancer and chemopreventive response to anti-inflammatory drugs. Gastroenterology (2011) 140(3):799–808. doi: 10.1053/j.gastro.2010.11.041 21115010 PMC3049858

[27] Zuijdgeest-van Leeuwen SD van den Berg JW Wattimena JL van der Gaast A Swart GR Wilson JH. Lipolysis and lipid oxidation in weight-losing cancer patients and healthy subjects. Metabolism (2000) 49(7):931–6. doi: 10.1053/meta.2000.6740 10910006

[28] Looney SW Hagan JL. Analysis of biomarker data: a practical guide. Hoboken, New Jersey, USA: John Wiley & Sons (2015).

[29] Fitzmaurice GM Laird NM Ware JH. Applied longitudinal analysis. Hoboken, New Jersey, USA: John Wiley & Sons (2012).

[30] Greenland S. Tests for interaction in epidemiologic studies: a review and a study of power. Stat Med (1983) 2(2):243–51. doi: 10.1002/sim.4780020219 6359318

[31] Crusz SM Balkwill FR. Inflammation and cancer: advances and new agents. Nat Rev Clin Oncol (2015) 12(10):584–96. doi: 10.1038/nrclinonc.2015.105 26122183

[32] Johnson DE O'Keefe RA Grandis JR. Targeting the IL-6/JAK/STAT3 signalling axis in cancer. Nat Rev Clin Oncol (2018) 15(4):234–48. doi: 10.1038/nrclinonc.2018.8 PMC5858971 29405201

[33] Schmitz KH Gehlert S Patterson RE Colditz GA Chavarro JE Hu FB. TREC to WHERE? Transdisciplinary research on energetics and cancer. Clin Cancer Res (2016) 22(7):1565–71. doi: 10.1158/1078-0432.CCR-14-1214 PMC4956346 26792261

[34] Friedenreich CM Neilson HK Woolcott CG Wang Q Stanczyk FZ McTiernan A. Inflammatory marker changes in a yearlong randomized exercise intervention trial among postmenopausal women. Cancer Prev Res (Phila). (2012) 5(1):98–108. doi: 10.1158/1940-6207.CAPR-11-0369 21982875

[35] Friedenreich CM O'Reilly R Shaw E Stanczyk FZ Yasui Y Brenner DR. Inflammatory marker changes in postmenopausal women after a year-long exercise intervention comparing high versus moderate volumes. Cancer Prev Res (Phila). (2016) 9(2):196–203. doi: 10.1158/1940-6207.CAPR-15-0284 26603740

[36] Del Rosso S Baraquet ML Barale A Defago MD Tortosa F Perovic NR. Long-term effects of different exercise training modes on cytokines and adipokines in individuals with overweight/obesity and cardiometabolic diseases: A systematic review, meta-analysis, and meta-regression of randomized controlled trials. Obes Rev (2023) 24(6):e13564. doi: 10.1111/obr.13564 37040899

[37] Edge SB Compton CC. The American Joint Committee on Cancer: the 7th edition of the AJCC cancer staging manual and the future of TNM. Ann Surg Oncol (2010) 17(6):1471–4. doi: 10.1245/s10434-010-0985-4 20180029

[38] Lohmann AE Dowling RJO Ennis M Amir E Elser C Brezden-Masley C. Association of metabolic, inflammatory, and tumor markers with circulating tumor cells in metastatic breast cancer. JNCI Cancer Spectr (2018) 2(2):pky028. doi: 10.1093/jncics/pky028 30035251 PMC6044231

[39] Uen YH Lu CY Tsai HL Yu FJ Huang MY Cheng TL. Persistent presence of postoperative circulating tumor cells is a poor prognostic factor for patients with stage I-III colorectal cancer after curative resection. Ann Surg Oncol (2008) 15(8):2120–8. doi: 10.1245/s10434-008-9961-7 18481151

[40] Lu CY Tsai HL Uen YH Hu HM Chen CW Cheng TL. Circulating tumor cells as a surrogate marker for determining clinical outcome to mFOLFOX chemotherapy in patients with stage III colon cancer. Brit J Cancer. (2013) 108(4):791–7. doi: 10.1038/bjc.2012.595 PMC3590657 23422758

