# Supplementary material for: The dose-response effect of aerobic exercise on inflammation in colon cancer survivors
Source: Front Oncol. 2023 Dec 12;13:1257767. doi: 10.3389/fonc.2023.1257767 (PMC10750999; doi:10.3389/fonc.2023.1257767)
Supplement: Supplementary file 1 [file DataSheet_1.docx]

Supplementary Material

## Supplementary Figures

**Supplementary Figure 1.** Flow of participants through the study


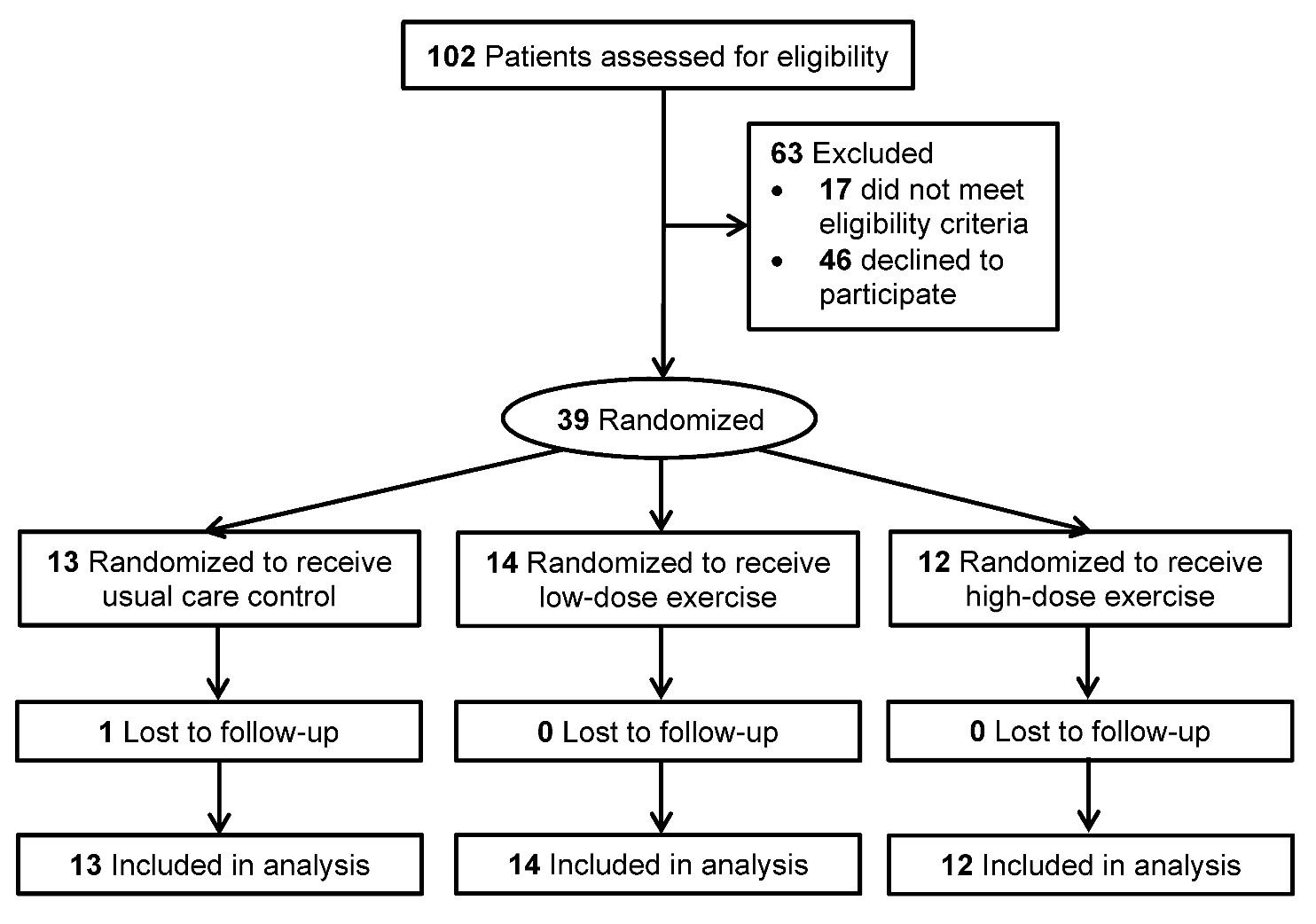


**
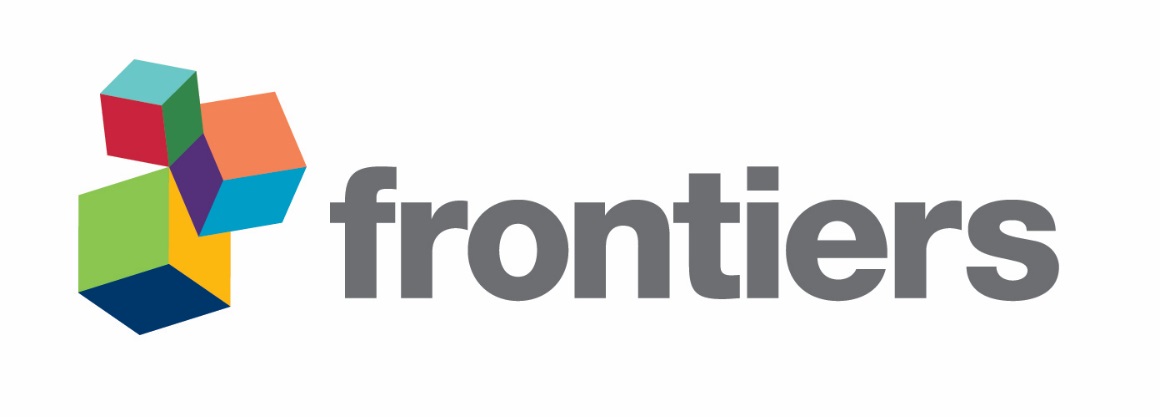
**
